# Behçet’s Disease Revealed by a Thrombosed Aneurysm of the Superior Mesenteric Vein: A Case Report

**DOI:** 10.7759/cureus.109733

**Published:** 2026-05-27

**Authors:** Omar Bahlaoui, Ahmed Bensaad, Jidal Sara, Anass Nadi, Imane Ben El Barhdadi

**Affiliations:** 1 Gastroenterology and Hepatology, Mohammed VI University of Health Sciences (UM6SS), Casablanca, MAR; 2 Gastroenterology and Hepatology, Mohammed VI International University Hospital, Casablanca, MAR; 3 Surgery, Mohammed VI University of Health Sciences (UM6SS), Casablanca, MAR; 4 Surgery, Mohammed VI International University Hospital, Casablanca, MAR; 5 Vascular Diseases, Mohammed VI International University Hospital, Casablanca, MAR

**Keywords:** angio-behçet, behçet’s disease, infliximab, portal vein thrombosis, superior mesenteric vein aneurysm, thrombosed venous aneurysm

## Abstract

Behçet’s disease is a rare multisystemic vasculitis that can involve arteries and veins of all sizes. Vascular involvement is among its most severe manifestations and usually presents as venous thrombosis, whereas venous aneurysms are exceptionally uncommon. We report the case of a 33-year-old woman admitted for abdominal pain, in whom imaging revealed a saccular thrombosed aneurysm of the superior mesenteric vein extending to the portal confluence. The patient had recurrent oral aphthosis, cutaneous lesions compatible with erythema nodosum, arthralgia, a positive pathergy test, and positive HLA-B51 testing. The thrombophilia work-up was negative, and ophthalmological examination was normal. The diagnosis of Angio-Behçet’s disease was established according to the International Criteria for Behçet’s Disease. She was treated with high-dose corticosteroid pulses followed by intravenous cyclophosphamide and oral prednisone. Anticoagulation was initiated with low-molecular-weight heparin, then switched to vitamin K antagonists and subsequently to apixaban because of INR instability. Serial Doppler ultrasound showed progressive regression of the aneurysm and thrombosis, with partial portal vein recanalization. Given persistent fibrotic retraction of the portal vein and concern for progression toward portal hypertension, infliximab therapy was planned. This case highlights an unusual presentation of Behçet’s disease revealed by a thrombosed superior mesenteric vein aneurysm and emphasizes the importance of early recognition, aggressive immunosuppressive treatment, multidisciplinary management, and close radiological follow-up.

## Introduction

Behçet’s disease (BD) is a chronic multisystemic vasculitis characterized by heterogeneous clinical manifestations, classically including recurrent oral and genital aphthosis, ocular inflammation, and systemic involvement. The disease is more prevalent in countries along the historical Silk Road, particularly in the Mediterranean basin, the Middle East, and East Asia. Vascular involvement occurs in up to 40% of cases and represents one of the most severe complications of the disease [1,2]. Venous manifestations, especially deep venous thrombosis, are considerably more frequent than arterial lesions. In contrast, aneurysms are uncommon and predominantly arterial, while venous aneurysms remain exceptionally rare. Superior mesenteric vein (SMV) aneurysms are among the rarest vascular manifestations described in BD, with only a limited number of cases reported in the literature [3,4]. Because abdominal symptoms are often non-specific, diagnosis may be delayed, potentially exposing patients to severe complications such as portal hypertension or thrombotic extension. We report a rare case of Angio-Behçet revealed by a thrombosed SMV aneurysmal lesion, highlighting the importance of considering BD in young patients presenting with unusual venous vascular abnormalities.

## Case presentation

A 33-year-old woman with no significant medical history, apart from a cholecystectomy 13 years earlier, was admitted for evaluation of intense abdominal pain evolving for three weeks, associated with nausea but without fever, vomiting, jaundice, or digestive bleeding. She reported a history of recurrent oral aphthosis and previous cutaneous lesions compatible with erythema nodosum, as well as episodes of oligo-arthralgia of the knees. No genital aphthosis was noted. She occasionally complained of blurred vision, but ophthalmological examination did not reveal uveitis or retinal vascularitis.

On admission, the patient was hemodynamically stable and afebrile. Laboratory evaluation showed elevated inflammatory markers (C-reactive protein (CRP) 35 mg/L), normal hemogram, and preserved hepatic and renal function.

**Figure 1:**
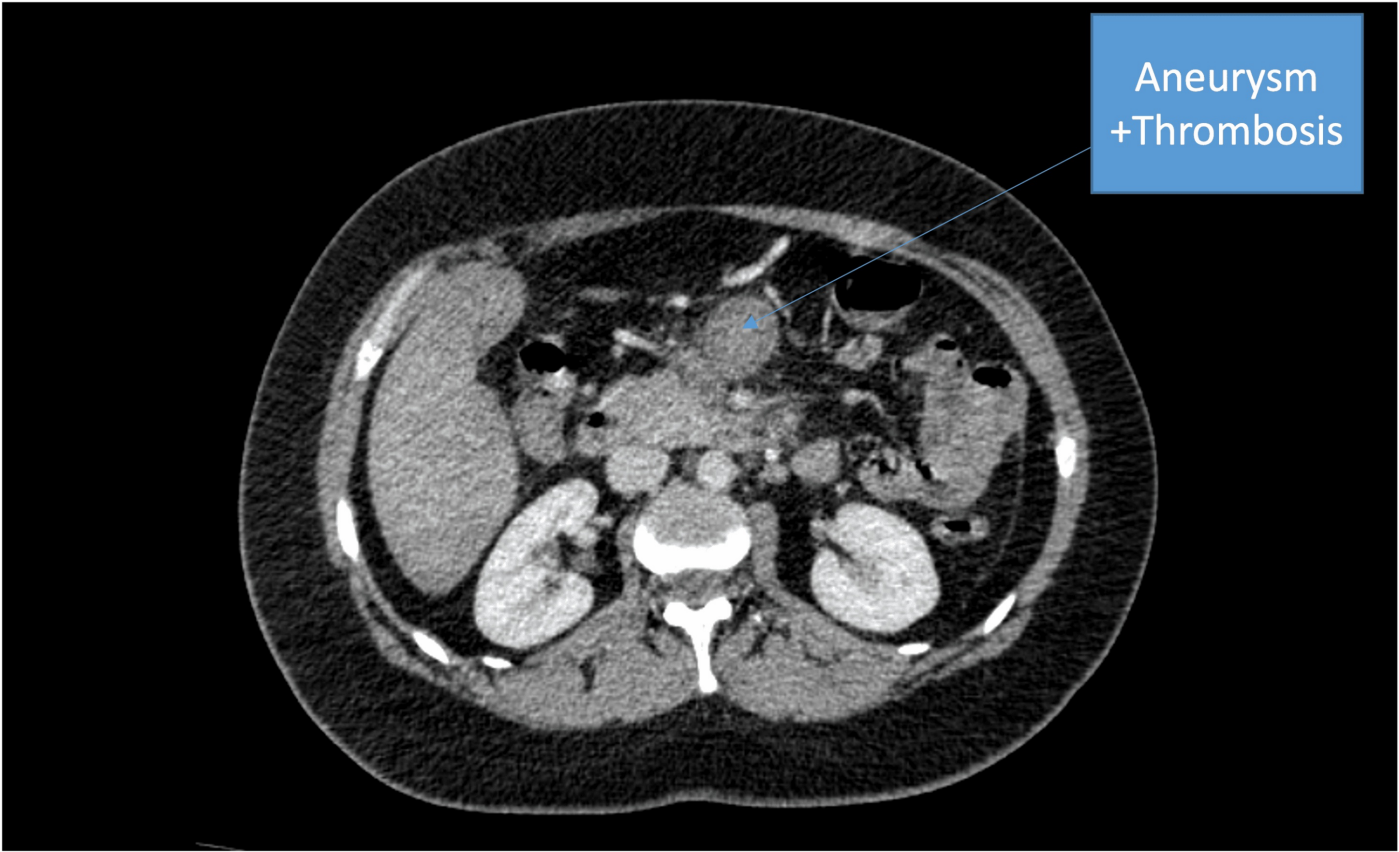
CT angiography showing a saccular, thrombotic aneurysm involving the superior mesenteric vein (SMV).

**Figure 2:**
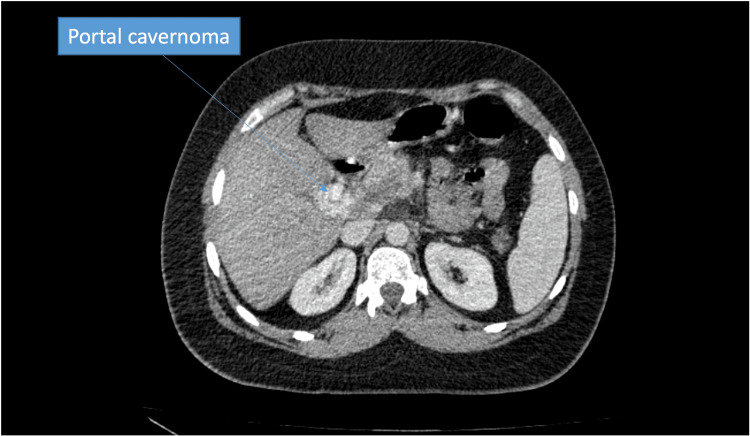
CT angiography showing a cavernomatous transformation of the portal vein.

**Figure 3:**
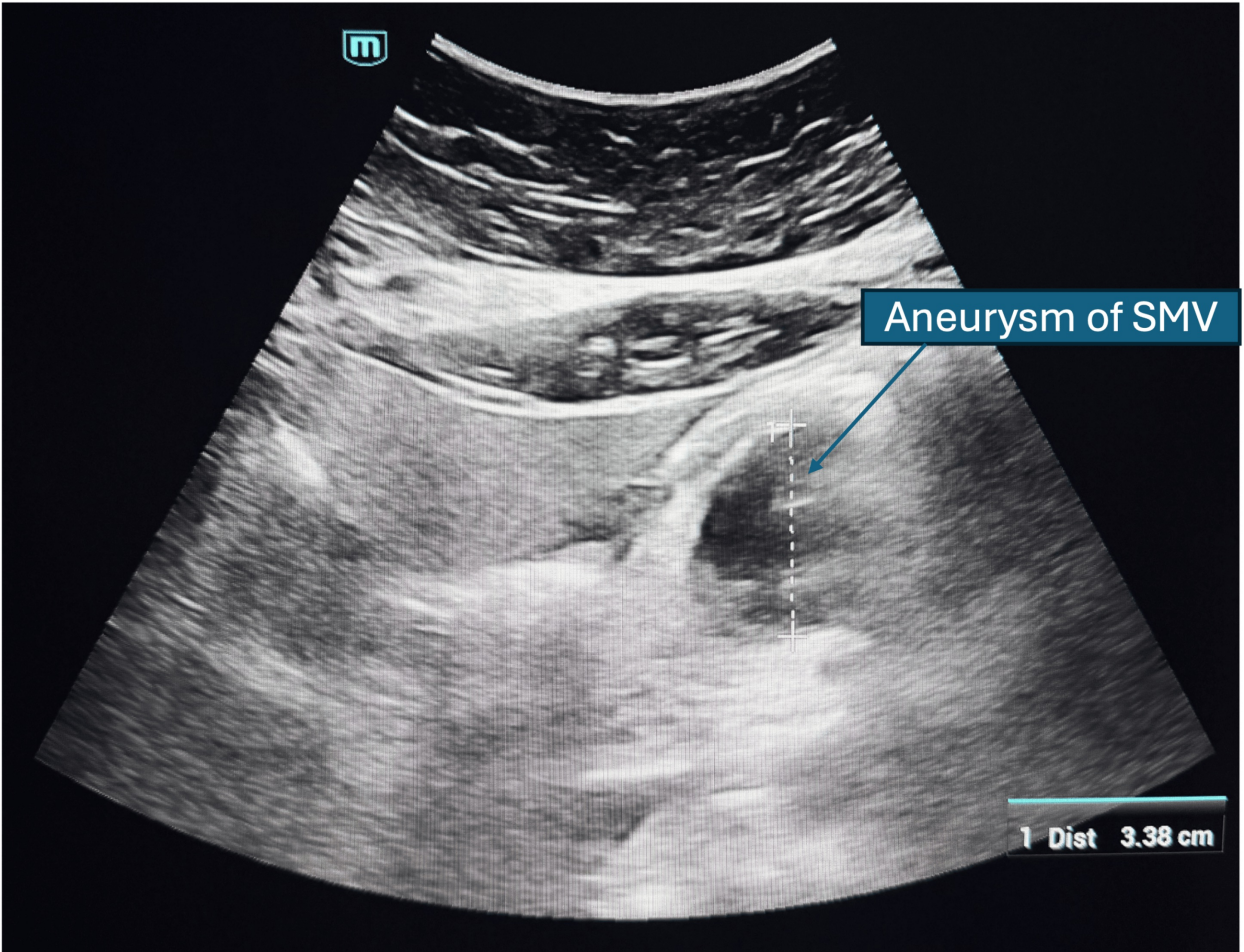
Serial Doppler ultrasound follow-up of the superior mesenteric vein (SMV) aneurysmal lesion and associated thrombosis under immunosuppressive therapy and anticoagulation, demonstrating progressive reduction in lesion diameter and partial portal vein recanalization. Baseline Doppler evaluation showed the absence of detectable flow within the aneurysmal sac.

On imaging, abdominal MRI demonstrated a saccular and thrombosed aneurysm of the SMV, extending to the portal confluence with perfusion disturbances of the liver. CT angiography confirmed a saccular, thrombotic aneurysm involving the SMV (Figure 1), with cavernomatous transformation of the portal vein (Figure 2) but no intestinal or splenic infarction (Figure 1). Doppler ultrasound corroborated the findings, showing an aneurysm measuring up to 33 mm (Figure 3).

Thrombophilia screening was negative (protein C, protein S, factor V Leiden, prothrombin gene mutation, antithrombin III, homocysteine). The pathergy test was positive. HLA-B51 typing was also positive.

According to the International Criteria for Behçet’s Disease (ICBD), the patient scored five points according to the 2014 ICBD: oral aphthosis (two points), cutaneous involvement compatible with erythema nodosum (one point), vascular involvement (one point), and positive pathergy test (one point). The diagnosis of Angio-Behçet’s disease was thus established.

The patient received high-dose intravenous corticosteroids (1 g/day for three days), followed on day 4 by a cyclophosphamide infusion (1 g IV). Corticosteroid therapy was continued orally with prednisone (60 mg/day). Monthly cyclophosphamide pulses were administered thereafter. She was initially treated with low-molecular-weight heparin (LMWH), followed by a switch to vitamin K antagonists.

**Figure 4:**
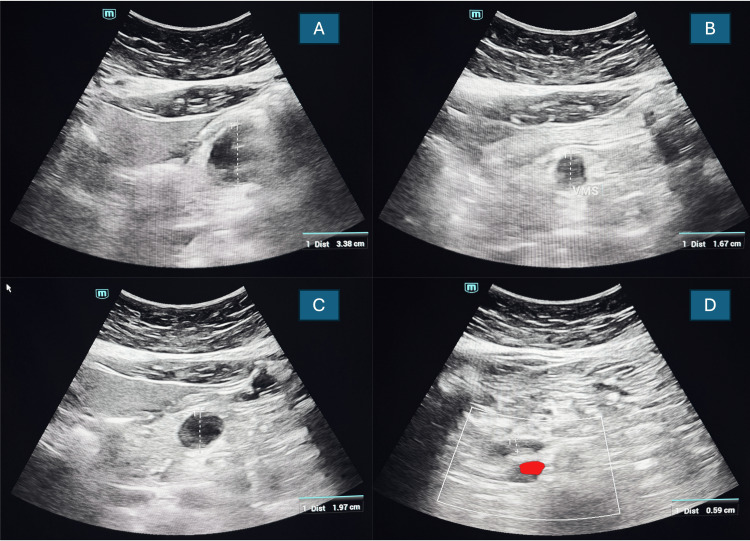
Serial Doppler ultrasound follow-up of the superior mesenteric vein (SMV) aneurysm and associated thrombosis under anticoagulation, showing progressive reduction in aneurysm diameter and partial portal vein recanalization. (A) Baseline examination (day 0), diameter 33 mm. (B) Follow-up at month 1, diameter 20 mm. (C) Follow-up at month 3, diameter 16 mm. (D) Follow-up at month 6, residual lesion measuring 6 mm with partial portal venous recanalization.

However, due to INR instability, anticoagulation was changed to Eliquis 5 mg twice daily. Serial Doppler ultrasound examinations performed at one month, three months, and six months demonstrated progressive regression of the lesion, with a decrease in aneurysm diameter from 33 mm initially to 20 mm, then 16 mm, and finally 6 mm (Figure 4, Table 1), along with partial re-permeabilization of the portal trunk. Given the persistence of fibrotic retraction of the portal vein on Doppler evaluation, the multidisciplinary team recommended escalation to biologic therapy with infliximab (5 mg/kg IV monthly) in order to prevent progression toward portal hypertension. Upper gastrointestinal endoscopy had not yet been performed at the time of manuscript preparation. Clinically, the patient showed resolution of abdominal pain, improvement of general condition, and normalization of inflammatory markers.

**Table 1:** Chronological summary of clinical presentation, investigations, treatment, and radiological evolution.

Time point	Clinical findings	Imaging findings	Treatment	Outcome
Admission	Abdominal pain	SMV thrombosed aneurysm 33 mm	IV steroids + LMWH	Clinical stabilization
Month 1	Improved pain	20 mm	Cyclophosphamide	Partial regression
Month 3	Stable	16 mm	Continued therapy	Further regression
Month 6	Asymptomatic	6 mm + partial recanalization	Consideration of infliximab	Favorable evolution

## Discussion

Vascular involvement in BD represents one of the most severe and life-threatening manifestations of this condition. While mucocutaneous and ocular signs are the classical hallmarks of BD, vascular disease can dramatically influence prognosis. It is estimated that vascular lesions occur in up to 40% of patients, most often within the first five years after disease onset [1]. Venous thrombosis is by far the most common vascular presentation, involving deep veins of the lower extremities, vena cava, or cerebral venous sinuses. Arterial involvement is less frequent but carries a higher risk of morbidity and mortality, mainly due to rupture of aneurysms in the pulmonary or aortic circulation [2]. Venous aneurysms, in contrast, are exceptionally rare, and aneurysms of the SMV are among the rarest vascular lesions described in BD [3,4].

The pathophysiology of vascular BD is complex and not yet fully understood. Several mechanisms are involved, including endothelial dysfunction, abnormal neutrophil activation, increased oxidative stress, and perivascular immune complex deposition [1,2]. These processes lead to damage of the vascular wall, with inflammatory infiltrates causing weakening and potential dilatation, explaining the formation of aneurysms even in venous structures, where pressure is physiologically lower compared to arteries. The presence of both aneurysm and thrombosis in our patient illustrates the dual spectrum of vascular BD: vessel wall weakening resulting in aneurysm formation, and a hyperinflammatory prothrombotic environment leading to thrombosis.

From a clinical perspective, our patient presented with abdominal pain, a non-specific symptom that can be related to many conditions such as pancreatitis, mesenteric ischemia, or portal thrombosis. The discovery of this thrombosed SMV lesion on imaging was therefore an unusual and striking finding. Very few similar cases have been reported, and in most, the diagnosis of BD was either already known or suspected due to other systemic signs [3,5]. In our case, the vascular lesion was the revealing manifestation, highlighting the importance of considering BD in the differential diagnosis of unexplained venous aneurysms or thrombosis in young patients.

Imaging plays a key role in the diagnosis and monitoring of vascular BD. In our patient, MRI, CT angiography, and Doppler ultrasound were complementary. MRI revealed the aneurysm, CT angiography further characterized the vascular lesion, showing thrombosis with cavernomatous transformation of the portal system, and Doppler ultrasound allowed dynamic follow-up and measurement of regression over time. Serial Doppler evaluations are particularly useful to monitor changes in size, thrombosis status, and portal vein involvement without repeated radiation exposure. Detailed vessel wall enhancement analysis and precise aneurysm neck measurements were not consistently available because the imaging studies were performed in a real-world clinical setting rather than using a dedicated vasculitis imaging protocol.

Regarding the diagnostic approach, BD remains a clinical diagnosis supported by classification criteria. The ICBD, initially proposed in 2006 and later validated in a large multinational cohort in 2014, provides a point-based system in which oral aphthosis, genital aphthosis, ocular involvement, cutaneous lesions, vascular involvement, and a positive pathergy test are weighted [6]. Our patient scored four points (oral aphthosis, erythema nodosum, vascular involvement, pathergy), fulfilling the threshold for BD. Of note, HLA-B51 testing was positive, which, although not required for diagnosis, supports the strong genetic association and has been linked with more severe disease and vascular complications.

Management of vascular BD is challenging, as there are no randomized controlled trials to guide therapy, and treatment strategies rely mainly on expert consensus and observational studies. Immunosuppressive therapy is considered the cornerstone of management in vascular forms. High-dose corticosteroids combined with cyclophosphamide are recommended in severe or life-threatening vascular involvement, particularly with large-vessel aneurysms [1,5]. In our patient, this regimen was initiated promptly, with significant improvement both clinically and radiologically.

The role of anticoagulation in vascular BD remains controversial. Some authors argue that anticoagulants have limited benefit, as BD-associated thrombosis is primarily driven by vessel wall inflammation rather than classic thrombophilia [1]. Moreover, anticoagulation carries the risk of aneurysmal rupture in cases of arterial involvement. In the present patient, anticoagulation was maintained because of the extensive thrombosis involving the superior mesenteric and portal venous system, associated with cavernomatous portal transformation, despite the recognized inflammatory pathophysiology of vascular BD and the ongoing controversy regarding anticoagulant therapy in this setting. Our patient was initially treated with LMWH, then transitioned to vitamin K antagonists. However, the use of direct oral anticoagulants in vascular BD remains off-label and is not specifically endorsed by current EULAR recommendations. Available evidence is currently limited to small observational studies and case series. In the present case, the decision to switch from vitamin K antagonists to apixaban was individualized because of persistent INR instability despite close monitoring, in a context of extensive mesenteric and portal venous thrombosis.

Biologic therapy, particularly anti-TNF agents such as infliximab, has revolutionized the management of refractory BD. Infliximab has shown efficacy in controlling severe vascular involvement, especially in patients with inadequate response to conventional immunosuppressants [7]. In our patient, despite regression of the aneurysm and thrombosis under corticosteroids and cyclophosphamide, persistent fibrotic retraction of the portal vein raised concern for portal hypertension. Therefore, escalation to infliximab was proposed because of persistent portal venous fibrotic retraction and concern for progression toward portal hypertension, although the documented vascular regression had already occurred under corticosteroids, cyclophosphamide, and anticoagulation. Azathioprine may also be considered as a maintenance immunosuppressive option in vascular BD.

This case contributes to the limited literature on venous aneurysms in BD and particularly highlights the rare involvement of the SMV. It underscores several important learning points: (1) BD should be suspected in young patients with unexplained venous aneurysms or thrombosis; (2) imaging is essential for diagnosis and follow-up; (3) aggressive immunosuppressive therapy can induce regression of venous aneurysms; (4) anticoagulation may be considered in extensive thrombosis, although it remains controversial; and (5) biologic therapy, especially infliximab, plays a crucial role in preventing progression and complications in severe vascular BD.

Ultimately, the prognosis of vascular BD depends on early recognition, appropriate immunosuppression, and careful long-term monitoring. Without timely treatment, patients are at high risk of recurrence, progressive aneurysmal disease, and life-threatening complications such as portal hypertension or rupture. Our patient’s favorable outcome illustrates the effectiveness of a multidisciplinary, stepwise approach involving corticosteroids, immunosuppressants, anticoagulation, and biologic therapy.

## Conclusions

We report a rare case of BD revealed by an unusual thrombosed SMV aneurysmal lesion with associated venous thrombosis. This presentation highlights the protean vascular involvement of BD and emphasizes the importance of considering BD in young patients with atypical venous aneurysms. The favorable radiological and clinical response observed under corticosteroids, cyclophosphamide, and anticoagulation highlights the importance of early aggressive immunosuppressive management in severe vascular BD. Because persistent portal venous fibrotic changes raised concern for progression toward portal hypertension, escalation to infliximab was planned as a preventive strategy requiring further longitudinal evaluation. Close radiological and clinical follow-up is essential to detect complications such as portal hypertension and ensure long-term disease control.
